# ‘Darkness as we face the unknown’: Biopsychosocial‐spiritual experiences of parents of children and young people with type 1 diabetes from the Middle East and North Africa (MENA) region

**DOI:** 10.1111/dme.70303

**Published:** 2026-03-29

**Authors:** Mariam Asaad, Haya Abu Ghazaleh, Vasiliki Tzouvara, Nada Aljohani, Shatha Alsayed, Jackie Sturt

**Affiliations:** ^1^ Florence Nightingale Faculty of Nursing, Midwifery and Palliative Care King's College London London UK

**Keywords:** diabetes, psychosocial, qualitative, type 1 diabetes

## Abstract

**Aim:**

Qualitative interviews were conducted as part of the development of a person‐reported outcome measure (PROM) to assess biopsychosocial‐spiritual outcomes of parents of children with type 1 diabetes (T1D) in the Middle East and North Africa (MENA) region. The aims were to (i) explore the biopsychosocial‐spiritual experiences of parents in the MENA region (ii) identify biopsychosocial‐spiritual constructs of the PROM.

**Methods:**

Two focus groups were conducted with *n* = 5 mothers of children with T1D (online), one focus group with *n* = 3 healthcare professionals (online) and one focus group with *n* = 6 policymakers (in person) interested in PROM development in Saudi Arabia. Four semi‐structured interviews were conducted with *n* = 4 fathers of children with T1D in Saudi Arabia (online). Interviews were audio‐recorded and transcribed verbatim using a thematic analysis approach.

**Results:**

Four main themes and several sub‐themes were identified: (1) *emotional well‐being:* (a) ‘Darkness as we face the unknown’: distress, worry and anxiety (b) familiarisation and empowerment: coping and optimism; (2) *bio‐physical well‐being:* (c) ‘I have to be vigilant’: insomnia and fear of hypoglycaemia; (3) *social impact and well‐being:* (d) stigma, sacrifice and support; and (4) *spiritual well‐being:* (e) ‘I leaned in on spirituality’: hope and acceptance.

**Conclusions:**

The themes and sub‐themes depict that diabetes has an impact on parents' biopsychosocial‐spiritual well‐being. All stakeholders had positive reflections in relation to the value of the development of a PROM as a tool for early detection of issues related to parents' biopsychosocial‐spiritual well‐being.

**What's new?**

**What is already known?**

- Parents of children and young people with T1D experience psychosocial distress.

**What has this study found?**

- There is a need for culturally sensitive biopsychosocial‐spiritual support for parents of children and young people with T1D.

**What are the implications of this study?**

- Healthcare professionals need to add depth and sensitivity to care by considering the ‘whole person’ when providing diabetes care.

## 1 INTRODUCTION

A review by Robert, Al‐Dawish, Mujammami and Dawish^1^ describes the rise in the rates of type 1 diabetes (T1D) in children in the Kingdom of Saudi Arabia (KSA) as a ‘soaring epidemic’. Recent data from the Diabetes Atlas report from the International Diabetes Federation (IDF) confirm this, where the number of individuals with T1D less than 20 years of age in KSA in 2022 is one of the highest in the world at 49,118 individuals, out of a total of 201,000 individuals.^2^

The diagnosis of a child or young person (CYP) with T1D is a life changing experience for the family^3^ as it involves constant monitoring of blood glucose levels to maintain glycaemic control and learning new skills such as how to give insulin injections or use a glucometer to measure blood glucose levels. Overnight a heavy responsibility is placed on the parents to prevent life threatening conditions such as hypoglycaemia or diabetic ketoacidosis (DKA); consequently, parents can find this an immense burden on their shoulders.^4^ Saudi mothers in a recent qualitative study expressed a need for psychological support to help them cope with feelings of distress and frustration when trying to support and co‐manage their children's condition; these feelings are mirrored by mothers from the United Arab Emirates (UAE) and Iran.^5^, ^6^, ^7^ However, biopsychospiritual aspects of diabetes care are not addressed as part of diabetes care in KSA.^8^

Person‐reported outcome measures (PROMs) enable us to better understand people's experiences of care and help to improve diabetes care and support. However, a recent systematic review of the outcomes of parents of children with T1D from the Middle East and North Africa (MENA) found that PROMs used to assess parental need did not consider the important spiritual and cultural context of the region.^9^ Previously developed PROMs to assess diabetes distress in parents of CYP with T1D, such as the Problem Areas In Diabetes (PAID) scale or the Parent Diabetes Distress Scale (DDS), were mainly developed within a Western context and therefore have not addressed important cultural values and beliefs that affect health and well‐being such as spirituality.^10^, ^11^

This qualitative study forms part of broader research to develop a reliable, valid and culturally sensitive tool to assess biopsychosocial–spiritual needs of parents with CYP with T1D in KSA. The aim of this study was to use interviews with parents and stakeholder input to identify appropriate domains and items important for the construction of a PROM for this population.

## 2 METHODS

### 2.1 Stakeholder and patient and public involvement (PPI)

Stakeholder and PPI allow their lived experience and knowledge to add value and shape research processes. Therefore, a steering group of parents (*n* = 4) of CYP with T1D in KSA was recruited through WhatsApp groups of which the lead researcher is a part. The steering group helped develop the interview topic guide, which helped guide the discussion for the focus group sessions.^12^

### 2.2 Design

This study used an exploratory, qualitative approach using focus groups to collect the data. Focus groups were used to elicit a wide variety of responses and encourage participants to exchange and share ideas and build on each other's responses.^13^ The paper reports the research according to the Consolidated Criteria for Reporting Qualitative Research (COREQ) guidelines.^14^

The themes from the qualitative data were then developed into constructs and domains, and then further refined by the research team into suitable items or questions for the construction of a PROM.

### 2.3 Participants

Purposive sampling was used to recruit parents of CYP with T1D (*n* = 9), healthcare professionals (HCPs) (*n* = 2) and policymakers (*n* = 6). The research was advertised on X (Twitter) and WhatsApp groups of which the lead researcher is part and in the endocrinology clinic at the Security Forces Hospital (SFH) in Riyadh, KSA. A pilot interview was conducted online with *n* = 2 mothers of CYP with T1D and *n* = 1 HCP to test the interview guide. Focus group interviews with policymakers and mothers started in October 2023, and four semi‐structured interviews were conducted with fathers of CYP in January 2024. Table 1 provides more details of the parents.

**TABLE 1:** Details of participants and methods of recruitment.

Participant group	Number of participants	Method of data collection	Method of recruitment	Reason for inclusion
Policymakers	*n* = 6	Focus group	The head of the ‘Value in Health’ organisation in KSA was a known contact of the lead researcher and they were recruited via email invitation	Their feedback would ensure the PROM was in line with the standards for PROM development in KSA
HCPs	*n* = 2 (Endocrinologist, diabetes educator)	Focus group	Recruited via social media platforms X(Twitter) and WhatsApp in KSA	Their reflections on the clinical importance of the PROM were an important part of the PROM development process
Mothers of CYP with T1D in KSA	*n* = 3	Focus group	Recruited from the paediatric endocrinology clinic at the SFH in Riyadh, KSA The lead researcher had previous connections with the paediatric endocrinology team from SFH from a previous study	Their perspectives would provide a wider view of the experiences of mothers from the region
Mothers of CYP with T1D from the UAE	*n* = 2	Focus group	Recruited via WhatsApp The lead researcher was part of online support groups for parents of CYP with T1D from KSA and the UAE and had access to these populations	
Fathers of CYP with T1D	*n* = 4	Semi‐structured interviews	Recruited from the paediatric endocrinology clinic at the SFH in Riyadh, KSA	The perspectives of fathers from KSA have not been included in previous research, despite the important role they play in helping to manage their child's condition

### 2.4 Characteristics of participants (parents)

#### 2.4.1 Inclusion criteria

- Parents of CYP with T1D living in KSA or Gulf Cooperation Council (GCC) region (Table 2).
- HCPs (endocrinologists, diabetes educators) working with CYP with T1D from paediatric endocrinology clinics in KSA.
- Policymakers from the Ministry of Health in KSA with an interest in PROM development.

### 2.5 Data collection

The focus group topic guide was informed by the biopsychosocial‐spiritual model.^15^ Participants' questions about their biopsychosocial‐spiritual experiences with their CYP's diabetes included: questions related to the (1) emotions they experience such as feelings of sadness or anxiety; (2) the bio‐physical impact of diabetes, such as sleep; (3) the social impact of diabetes, such as stigma and support; (4) the spiritual well‐being, such as spiritual methods of coping. In addition, some questions related to the value of PROMs were included in the interview schedule of the HCPs and policymakers.

The topic guide was then piloted on a group of parents *n* = 4 and HCPs *n* = 1 to check that the questions yield responses related to the objectives of the focus groups. No changes were made after the pilot.

An initial focus group was conducted face‐to‐face in English (as this was the native language of one of the participants) at the office of the six policymakers. A second online focus group session was conducted with two HCPs (to allow them to participate after their working hours). Two further online focus group sessions were conducted in Arabic with the three mothers from KSA and one in English with the two mothers from the UAE. All focus groups were facilitated by the lead researcher (MA) and were moderated by members of the research team (NA) and (SA), who are bilingual and fluent in both languages. Semi‐structured individual interviews were conducted online in Arabic by the lead researcher (MA) with the fathers, as culturally they were not comfortable with the idea of talking about their biopsychosocial‐spiritual experiences in a group setting.

The lead researcher (MA) is a Saudi nurse by background and a mother to two children with T1D and has been part of peer support groups of parents of CYP with T1D in KSA and the GCC. As a parent of two children with T1D the power dynamic in the relationship between the lead researcher (MA) and the participants was more equal which is an important part of ensuring the trustworthiness of the data.^16^ Another criterion of trustworthiness was MA's prolonged experience with ‘diabetes’ the phenomena of research.

**TABLE 2:** Characteristics of parents and their CYP with T1D.

Participant number	Type of participant	Sex of CYP with T1D	Age of CYP at diagnosis	Duration of diabetes of CYP	Nationality	City of residence
P1	Parent (mother)	Boy	6 years	1.5 years	Saudi	Riyadh, KSA
P2	Parent (mother)	Boy	12 years	2 years	Saudi	Riyadh, KSA
P3	Parent (mother)	Girl	3 years	13 years	Saudi	Riyadh, KSA
P4	Parent (mother)	Boy	2 years	12 years	USA	Dubai, UAE
P5	Parent (mother)	Girl	7 years	3 years	Egyptian	Dubai, UAE
P6	Parent (mother)	Girl	6 years	5 years	Saudi	Riyadh, KSA
P7	Parent (father)	Boy	3 years	1 year	Saudi	Qunfutha, KSA
P8	Parent (father)	Girl	6 years	5 years	Saudi	Riyadh, KSA
P9	Parent (father)	Boy	5 years	2 years	Saudi	Riyadh, KSA
P10	Parent (father)	Girl	6 years	1 year	Saudi	Riyadh, KSA

Although no member checking was conducted, information from the focus groups/interviews was used to construct PROM items within a survey developed using Qualtrics.^17^

### 2.6 Method of data analysis

Transcripts from the audio recordings were transcribed verbatim and translated from Arabic to English by the MA and HA who are fluent in both languages and were coded using NVIVO software (Version 14),^18^ some transcripts were coded independently by two other researchers (HA, VT) to enhance dependability and credibility of data analysis. Coding was checked by (NA, SA). An inductive and deductive thematic analysis approach by Braun and Clarke^19^ guided the analysis process of the qualitative data whereby the analysis was data‐driven.

### 2.7 Ethical considerations

Ethical approval was sought in October 2023 by (a) Kings College London (KCL) with reference number HR/DP‐23/24‐39261 and (b) the SFH with reference number KACST, KSA: H‐01‐R‐069. Verbal consent for participation was obtained prior to beginning the focus group or the individual semi‐structured interview, and permission was sought prior to audio recording.

## 3 RESULTS

**FIGURE 1:**
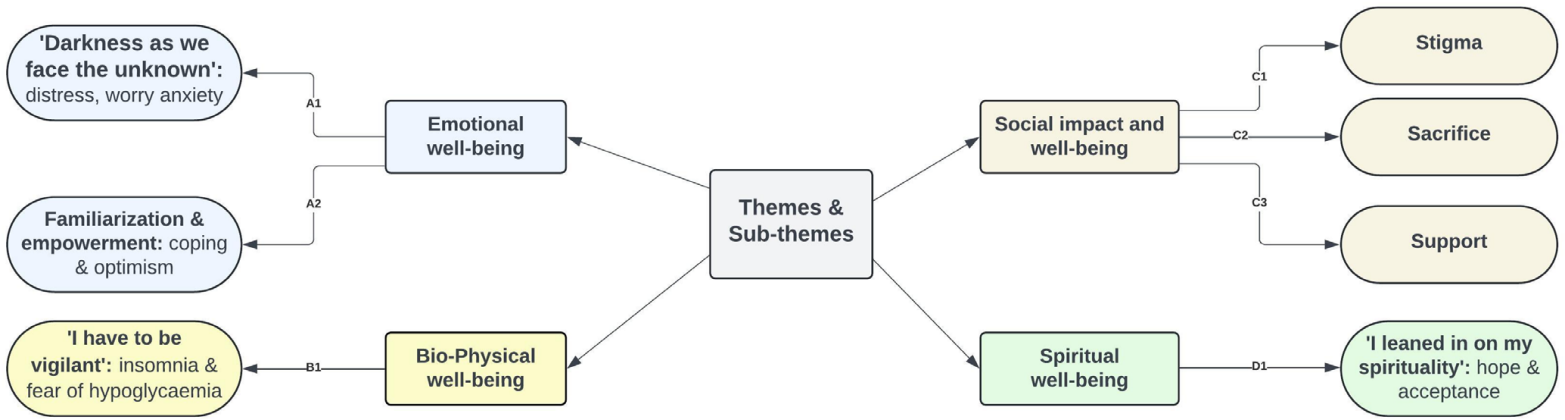
Themes and sub‐themes.

Four main themes: (1) emotional well‐being, (2) biophysical well‐being, (3) social impact and well‐being and (4) spiritual well‐being and several sub‐categories or constructs were identified from the qualitative interviews. These are depicted in Figure 1 below.

### 3.1 Theme a: Emotional well‐being

#### 3.1.1 Sub‐theme a 1:‘Darkness as we face the unknown’: Distress, worry and anxiety

Parents described the period after diagnosis as a period of darkness where they faced the unknown. In Islamic‐based cultures, the symbolism of light and darkness refers to broader concepts; darkness is associated with lack of knowledge, misguidance and disbelief.^20^ On the other hand, light is associated with knowledge, truth and guidance.^21^

##### Distress

Mothers linked discovering their child's diagnosis to feeling a sense of trauma and some reported excessive crying, while fathers found the unexpectedness of the diagnosis shocking:

Thank God, it was a shock, I really hoped I could delay it. But thank God for everything. I was shocked. (Father from KSA P8)

Trying to identify the cause of diabetes also caused parents to have distress and a feeling of guilt. For example, some parents worried about whether not breastfeeding or controlling their child's nutritional intake had contributed to their condition:

You just feel exhausted from the constant thinking about it, what could I have done better? This that and the other… (Mother from UAE P4)

A sense of feeling alone was expressed particularly by mothers and of not being ready to face the responsibility of managing their child's diabetes on discharge. HCPs attributed parental distress to realising the burden of the new commitments and being at beginning of a steep learning curve.

##### Worry and anxiety

Parents discussed their experiences of anxiety and worry, in relation to the various dimensions of both their children and their lives. For example, one father shared how they delayed future plans to have more children because they were anxious about another child having the condition. In addition, there was also anxiety about the cost of diabetes technology as some parents had to self‐fund continuous glucose monitoring (CGM). A mother from the UAE also shared her frustration with access to healthcare insurance:

We pay for the devices out of pocket, cause it's cheaper than if were to get a policy that covers it all. People really struggle with that. It makes caretaking even more challenging. (Mother from UAE P4)

Parents also identified worries about what might happen in the future. For example, some were constantly worried about the risk of hypoglycaemia, others about access to insulin during times of political unrest. Parents also worried about problems related to their child's ability to go to school or have a family. HCPs were aware of these fears which were brought up by worried parents in clinic:

The family are worried about the future, for girls if they will get married or not get married, if she will become pregnant, and if the pregnancy will be high risk or not. (Endocrinologist from KSA P17)

HCPs acknowledged the high levels of anxiety in parents, their need for emotional support, and spoke about referring them to emotional support platforms.

#### 3.1.2 Sub‐theme A2: Familiarisation and empowerment: Coping and optimism

Parents shared how they had become more confident over time with managing their child's diabetes and had become more familiar with symptoms of hyper/hypoglycaemia. Diabetes technology also played a big role in helping to empower parents to reach a stage of normalisation. For example, some reflected how glucose monitoring technology allowed them to monitor their children remotely whilst at school. Some parents expressed confidence and optimism that their child would become independent in time:

Like the beginning has passed, the future where he relies on himself will come easily and will pass. (Father from KSA P7)

Parents identified different methods of coping with the stress of diabetes. This included investing in themselves and having therapeutic outlets such as writing, or gardening. Parents felt empathy for families with a new diagnosis and felt empowered by providing them with support:

I saw a mother whose son was diagnosed a week previously, she really needed emotional support. I was happy to support her, (Mother from KSA P2)

### 3.2 Theme B: Bio‐physical well‐being

#### 3.2.1 Sub‐theme B1: ‘I have to be vigilant’: Insomnia and fear of hypoglycaemia

The impact of the child's condition on the parents' sleep was experienced by several parents. These feelings arose at night as they had to monitor their child's glucose levels to avoid hypoglycaemia. Some parents would take turns to stay awake or set alarms to check levels. Some found it challenging to wake the child up to treat a hypo. Waking up was another concern as one father described:

At night, I wouldn't sleep, I would stay awake until they woke up, because it was new to us, and we were afraid of hypos ….We were afraid we wouldn't wake up to the alerts. (Father from KSA P10)

### 3.3 Theme C: Social impact and well‐being

#### 3.3.1 Sub‐theme: Stigma, sacrifice and support

##### C1: Stigma

Parents reflected on their experiences with social stigma and how a lack of understanding about diabetes led to public misconceptions. In fact, a mother recalled her husband being anxious about her son taking injections in public for fear of what people may think. Others spoke about the perception by some people that diabetes could be transferred to others:

However, the social conception that this is a contagious disease remains with people and is even in young students. (Mother from KSA P3)

Whilst many parents had some scientific understanding around their child's diabetes, wider families could believe that the ‘evil eye’ was the reason for the condition and pressured parents to visit a religious scholar for help:

Our families said the boy has suffered from envy or the bad eye, or something like that. So, take him to a Sheikh (religious scholar). This kind of talk upset me because it's not based on science. (Father from KSA P7)

##### C2: Support

###### Support from schools

There were conflicting reflections of parents when it came to their child's school. Some schools regarded the pupil's diabetes as a burden. For example, one school would only accept their child if the mother was on site to administer insulin injections and treat hypoglycaemia throughout the day.

Some schools were supportive and allowed pupils with T1D toilet breaks or permitted the use of mobile phones to monitor glucose readings in class. However, teachers' lack of awareness about diabetes meant that students sometimes got into trouble for CGM alerts going off in class, or that they were not allowed to go to the toilet:

This year the science teacher would refuse to let him go to the toilet. He said “You're using your diabetes as an excuse.” (Mother from KSA P2)

HCPs also acknowledged the challenges parents face with their child's schools, and how governments schools are particularly lacking in providing support for people with chronic long‐term conditions, which forced some into private education.

###### Support from family and friends

Family support was important to parents, especially for parents who had other family members with diabetes as they could provide diabetes‐specific advice. One father reflected on how his wife's sister had T2D and was the best source of support for both his wife and daughter. Another father reflected on how his father, who had T2D, was constantly sharing advice. A mother expressed her gratitude to her friends who had children with T1D, who would coach her through treating a hypo:

I had friends. They would stay up with me at night if I needed them. Like the hypos at night. (Mother from UAE P5)

On the other hand, some parents reflected on how their family did not understand diabetes or the need for insulin. Some relatives were scared of taking responsibility of caring duties for their child with diabetes. One described her mother's attitude:

Don't bring him to me, I don't know what to do with him. (Mother from KSA P1)

##### C3 sacrifice

When parents were not supported, they were forced to sacrifice other aspects of their lives. Mothers, in particular, felt unable to delegate caregiving diabetes management activities to others. Sometimes they had to make changes to their careers or miss out on social events because of needing to always be there to give injections:

You feel you need to take care of them, so you'll take care of your things last. (Mother from UAE P4)

###### Support from HCPs

Reports of the support provided by HCPs were also a mixture of positive and negative experiences. Some of the parents interviewed received very good support:

The educator was amazing; she taught me everything from sick day management to carb counting. I had everything I needed. (Mother from UAE P4)

On the other hand, some parents were frustrated by the lack of empathy of the HCPs. Whilst parents wanted as much information as possible on how to manage their child's condition, not all HCPs provided the guidance that parents needed:

What was sad, is that the HCP team was not supportive, I would try to drag the information out of them to help me, to get me out of the darkness I was living in. (Mother from KSA P3)

###### Online support

Social media platforms such as WhatsApp and Telegram were a source of peer support for parents. These participants explained how they sought out psychological support from their peers, who could identify with them:

We entered the world of T1D and the groups on Telegram and thank God things are going ok. (Father from KSA P7)

An online anonymous psychological support application helped one father and his wife by providing advice to support their emotional well‐being. HCPs also reflected on how they would refer parents with anxiety to the online anonymous psychological support platforms.

### 3.4 Theme D: Spiritual wellbeing

#### 3.4.1 Sub‐theme D1: ‘I leaned in on spirituality’: Hope and acceptance

After the diagnosis of their child with T1D some parents sought spirituality through prayer, and sometimes yoga. Some reflected that their spirituality and faith provided emotional support for managing her child's condition: as one mother described in an excerpt:

If it wasn't for God, my daughter and I would have been lost. A sensation of calm and tranquility overcomes you; it gives you emotional support without you feeling it or being aware of it. (Mother from KSA P3)

The association between the child's diagnosis with T1D and the concepts of suffering and penance was highlighted by participants, and their child's condition was seen as a test of their patience and faith. When parents attributed their child's diabetes diagnosis to God's will and fate, it also made it easier to come to terms with it.

HCPs also reflected on how parents were able to better accept their child's condition by putting it in God's hands and how faith played a role in providing hope:

The religion is important because it allows one to have faith in their fate and destiny, it gives hope. (Endocrinologist from KSA P17)

However, others explained how being religious or spiritual did not necessarily help them deal with difficult situations or practical challenges of diabetes management.

## 4 DISCUSSION

The results provide unique insights that parents of CYP with T1D from the MENA region experience biopsychosocial‐spiritual distress whilst caring for their child. Moreover, they highlighted the role spirituality can play in parents reaching a stage of coping and acceptance of their CYP's condition. These findings are confirmed by a recent systematic review that explored spirituality among parents of children with life‐limiting or threatening chronic conditions: the review confirmed that parents identify ‘spirituality’ as a source of strength and a method for emotional and social coping.^22^ Moreover, it also identified the potential for spirituality to be a ‘process of struggle’, which resonates with the themes of penance and divine tests that parents in this study described.^22^ The themes and sub‐themes that emerged from the analysis of the interviews were transformed into domains and constructs that were used as a basis for the development of the PROM.

The findings show the cross‐cultural similarities in the negative emotions such as emotional distress, anxiety and worry that parents of CYP with T1D experience across the world in countries such as Sweden,^23^ the United Kingdom (UK),^24^ and New Zealand.^25^ Worrying about their children also led to poor sleep, and diabetes management led to some parents setting alarms to check glucose levels throughout the night to respond to alerts at all hours from their child's CGMs. This is similar to the experiences of parents from Sweden who reported they were *‘living on a timer’* to monitor their child's blood sugar levels at night.^3^

In addition, the social lives of parents were impacted by stigma and societal misconceptions about diabetes. Indeed, some parents also had to make social sacrifices because they did not have caregiving support. This is also similar to a study of mothers in Iran where extended families were afraid to provide caregiving support due to the complexity of care for a child with diabetes.^26^ Over time, some parents had to give up their careers because they could not manage caring for a CYP with T1D and the lack of support from their workplace; this is similar to the experiences of parents of children with life‐limiting conditions in Australia, where they felt unsupported to return to the workplace.^27^ A number of parents also had negative social support experiences from their child's school or HCPs. Whilst parents from the West experienced good support from school, some parents from Scotland felt the decision‐making during clinical consultations was one‐way and their needs were unmet by HCPs.^28^

Parents also spoke about the facilitators to coping better with their child's condition. For example, positive support from family, friends and peers on social media groups was very helpful to parents. In addition, spirituality helped parents come to terms with their CYP's condition and reach a stage of acceptance. Moreover, it gave parents hope and provided the prospect of a better future for their CYP. This is a similar finding to the experiences of parents of children with other chronic long‐term conditions in Brazil and Australia where spirituality and religious practises gave parents a sense of hope for their children with chronic conditions.^27^, ^29^

### 4.1 Strengths and limitations

Unlike previous studies conducted in the MENA region that mainly focused on mothers' experiences,^5^, ^6^, ^30^ a strength of this study was the inclusion of experiences of fathers from KSA. Credibility was strengthened by including the perspectives of several stakeholders such as policymakers, HCPs and the voice of the most important stakeholders *‘parents’* to ensure they have an impact on the research.^31^ A limitation was the number of HCPs who participated in this study, and this was due to difficulties in recruitment of this group. In addition, this study included participants from large urban areas within KSA and the UAE with only one parent from a rural area. Another potential caveat is that fathers were not happy to participate in a focus groups which may have or may not have revealed more information.

### 4.2 Implications for practice

Current guidance for diabetes care has a significant gap in spiritual care.^32^ A more integrated treatment approach should focus on conversation models, screening, assessment and spiritual care interventions.^32^ Further studies to explore what these interventions may look like are necessary.

This study has highlighted that schools can cause both parents and children more reason for emotional distress. Therefore, there is a need for education authorities, health authorities, schools and parents to collaborate to ensure the safety of the student with T1D at school. The International Society for Pediatric and Adolescent Diabetes (ISPAD) position statement can help provide detailed guidance on how this can be achieved.^33^

This PROM is relevant to parents from this population; however, through further testing and cultural validation it may be applicable to parents from other regions of the world.

## 5 CONCLUSIONS

The qualitative findings from this study and the findings from our systematic review^9^ have highlighted the lack of psychosocial support for parents in the MENA region; however, the emerging theme of *‘spirituality’* illustrates the importance of spirituality to this population.

This study shows the psychosocial‐spiritual gap in the assessment of parents' needs in clinical care in the region. Thus, it affirms the need for the translation of these experiences from the qualitative interviews into a deeper, more sensitive PROM to help HCPs consider the whole person.

## CONFLICT OF INTEREST STATEMENT

No potential conflict of interest was reported by the authors.
